# The Role of Social Support in Telehealth Utilization Among Older Adults in the United States During the COVID-19 Pandemic

**DOI:** 10.1089/tmr.2021.0025

**Published:** 2021-11-09

**Authors:** Grace S. Chung, Chad S. Ellimoottil, Jeffrey S. McCullough

**Affiliations:** ^1^ Department of Health Management and Policy, University of Michigan School of Public Health, Ann Arbor, Michigan, USA.; ^2^ Department of Urology, University of Michigan, Ann Arbor, Michigan, USA.

**Keywords:** telehealth, social support, COVID-19, aging

## Abstract

**Background:** Older adults may experience a significant digital divide and need support with using technology to transition to telehealth. This study examines the role of social support for telehealth utilization among older adults during the COVID-19 pandemic.

**Materials and Methods:** We used data from the COVID-19 Sample Person Interview to the National Health and Aging Trends Study. Using logistic regression, we measured the association between telehealth utilization and social support.

**Results:** Nearly one in five respondents used telehealth during the COVID-19 pandemic (weighted %: 20.6 [585/3188]). Currently living with family or friends and receipt of technical support were associated with telehealth utilization. Among residents of an assisted living facility, those who received communications technology support from the facility were more likely to use telehealth.

**Conclusion:** Health care providers and policies should aim to reduce barriers to telehealth among older adults, with efforts such as digital literacy support and training.

## Introduction

Older adults face barriers to health care access during the COVID-19 pandemic. Since COVID-19, many providers have become more accessible through telehealth, yet older adults use less telemedicine.^1,2^ They may experience a significant digital divide and need support with using technology to transition to telehealth.^3^ This study examines the role of social support for telehealth utilization among older adults during the COVID-19 pandemic using the National Health and Aging Trends Study (NHATS) COVID-19 supplement.

## Methods

We used data from the COVID-19 Sample Person Interview to NHATS and demographic data from Rounds 9 and 10.^4^ These data were from 3,188 participants aged 70 years and older. COVID-19 questionnaires were mailed from the end of June 2020 through October 2020, and collection continued through January 2021.^5^ The unweighted response rate was 83.5%. NHATS is sponsored by the National Institute on Aging (grant number NIA U01AG32947) and was conducted by Johns Hopkins University.

We measured the association between telehealth utilization and social support, conditional upon respondent characteristics. Our first model only included respondent characteristics, specifically age, gender, race/ethnicity, income, and presence of any chronic conditions associated with high risk for severe COVID-19. The second model controls for technological skill, measured by frequent use of e-mails or texts. The third model incorporates social support metrics: currently living with family or friends and receipt of support with learning a new technology or program to go online. Finally, among residents in an assisted living facility, we looked at communications technology support from the facility as a predictor. All models were estimated by logistic regression using the survey's weights.^6,7^ Marginal effects and standard errors (SEs) are reported.* This study was exempt from institutional review board review.

## Results

Nearly one in five respondents used telehealth during the COVID-19 pandemic (weighted %: 20.6 [585/3188]) (Table 1). Model 1 shows that compared with 70–75-year olds, the probability of telehealth utilization is lower by 6 percentage points (SE: 3) for those aged 80–85 years and by 7 percentage points (SE: 2) for those aged 85 years and older. Compared with older adults with low income (≤$40,125), the likelihood of telehealth utilization is higher by 7 percentage points (SE: 2) for those with middle ($40,125–$85,525) and by 14 percentage points (SE: 2) for those with high (>$85,525) income. Upon adding technological skill in Model 2, the age effects are no longer statistically significant. However, technology skill increases the probability of telehealth use by 11 percentage points (SE: 2).

**Table 1.** Participant Characteristics by Telehealth Use

Characteristic	No. (%)		
	Total	Telehealth use	No telehealth use
Total	3,188 (100.0)	585 (20.6)	2,603 (79.4)
Age, years			
≥70 and <75	543 (100.0)	128 (23.9)	415 (76.1)
≥75 and <80	932 (100.0)	200 (22.6)	732 (77.4)
≥80 and <85	755 (100.0)	118 (16.4)	637 (83.6)
≥85	958 (100.0)	139 (15.1)	819 (84.9)
Gender			
Male	1,343 (100.0)	252 (20.9)	1,091 (79.1)
Female	1,845 (100.0)	333 (20.4)	1,512 (79.6)
Race/ethnicity			
White, non-Hispanic	2,426 (100.0)	438 (20.3)	1,988 (79.7)
Black, non-Hispanic	527 (100.0)	92 (20.1)	435 (80.0)
Other	235 (100.0)	55 (23.8)	180 (76.2)
Income ($)			
≤40,125	1,590 (100.0)	227 (15.5)	1,363 (84.5)
>40,125 and ≤85,525	944 (100.0)	191 (21.8)	753 (78.2)
>85,525	654 (100.0)	167 (28.4)	487 (71.6)
Has a high-risk chronic condition^a^			
No	472 (100.0)	66 (17.3)	406 (82.7)
Yes	2,716 (100.0)	519 (21.3)	2,197 (78.7)
E-mails or texts often			
No	2,026 (100.0)	274 (14.6)	1,752 (85.4)
Yes	1,162 (100.0)	311 (28.1)	851 (71.9)
Currently living with family or friends			
No	1,047 (100.0)	140 (15.3)	907 (84.7)
Yes	2,141 (100.0)	445 (22.8)	1,696 (77.2)
Learned a new technology or program to go online^b^ with someone's help			
No	2,409 (100.0)	387 (18.6)	2,022 (81.4)
Yes	470 (100.0)	149 (32.2)	321 (67.8)
Assisted living facility helped residents keep in touch with family or friends online			
No	76 (100.0)	11 (13.8)	65 (86.2)
Yes	139 (100.0)	34 (28.4)	105 (71.6)

^a^ High-risk chronic conditions include heart disease, hypertension, history of stroke, cancer, diabetes, or lung disease.

^b^ This includes learning to use a smartphone, computer, or iPad or a program such as Zoom or FaceTime.

Model 3 incorporates social determinants. The likelihood of telehealth utilization is 5 percentage points (SE: 2) higher for those currently living with family or friends and 8 percentage points (SE: 2) higher for those receiving technical support. The receipt of technical support is a significant predictor conditional on living with family or friends (0.09, SE: 0.03, *p* = 0.003), but is no longer significant conditional on neither living with family or friends nor residing in an assisted living facility (0.08, SE: 0.04, *p* = 0.06) (not reported in Tables). Among residents of an assisted living facility, the receipt of communications technology support from the facility increases the probability of telehealth use by 14 percentage points (SE: 7) (Table 2).

**Table 2.** Results of Multivariable Logistic Regression Analyses Examining Telehealth Use as the Outcome

Characteristic	Model 1 Marginal probability (SE)	Model 2 Marginal probability (SE)	Model 3^a^ Marginal probability (SE)	Model 4^b^ Marginal probability (SE)
Currently living with family or friends			0.05 (0.02)^*^	
Learned a new technology or program to go online with someone's help			0.08 (0.02)^***^	
Assisted living facility helped residents keep in touch with family or friends online				0.14 (0.07)^*^
E-mails or texts often		0.11 (0.02)^***^	0.10 (0.02)^***^	0.10 (0.07)
Age (years)				
<75	Referent	Referent	Referent	Referent
≥75 and <80	−0.01 (0.02)	−0.004 (0.02)	−0.005 (0.02)	0.24 (0.17)
≥80 and <85	−0.06 (0.03)^*^	−0.04 (0.03)	−0.04 (0.03)	−0.18 (0.15)
≥85	−0.07 (0.02)^**^	−0.04 (0.02)	−0.03 (0.02)	−0.07 (0.15)
Female gender	0.02 (0.02)	0.007 (0.02)	0.008 (0.02)	−0.17 (0.06)^**^
Race/ethnicity				
White, non-Hispanic	Referent	Referent	Referent	Referent
Black, non-Hispanic	0.03 (0.03)	0.04 (0.03)	0.04 (0.03)	−0.06 (0.11)
Other	0.07 (0.04)	0.07 (0.04)	0.06 (0.04)	−0.15 (0.07)^*^
Income ($)				
≤40,125	Referent	Referent	Referent	Referent
>40,125 and ≤85,525	0.07 (0.02)^**^	0.05 (0.02)^*^	0.04 (0.02)	−0.06 (0.07)
>85,525	0.14 (0.02)^***^	0.10 (0.03)^***^	0.07 (0.02)^**^	0.08 (0.07)
Has a high-risk chronic condition	0.06 (0.03)^*^	0.06 (0.03)^*^	0.07 (0.03)^*^	0.05 (0.10)
No. of observations	3,188	3,188	3,188	215

^*^ *p* < 0.05; ^**^p < 0.01; ^***^p < 0.001.

^a^ Missing values of the “learned a new technology or program to go online with someone's help” imputed using multiple random imputation.

^b^ Limited to study participants in an assisted living facility.

SE, standard error.

## Discussion

We find that nearly one in five older U.S. adults used telehealth during the study period. Affluent and tech-savvy seniors are much more likely to use telemedicine. Respondents' living arrangements (living with family or friends) and receipt of technical support from family or friends are also important predictors, together accounting for more than half of telehealth utilization. Facility staff appear to play a similar role for respondents in assisted living facilities.

Previous studies have found limited telemedicine adoption among the elderly. Data from the National Poll on Healthy Aging showed that only 4% of adults aged 50–80 years had ever participated in a telehealth visit in May 2019.^8^ A study by Lam et al. of 4,525 older adults in the United States using 2018 NHATS data estimated that 38% were not ready for video visits, primarily due to inexperience with technology.^9^ Our study highlights the important role of social support for telehealth utilization among older adults who have limited experience and comfort with technology.

A major limitation of the study is its cross-sectional design with most of the questionnaires completed in July and August 2020. Thus, we cannot address how patterns changed as the pandemic, and experience with telemedicine evolved over time. Furthermore, our ability to assess causal relationships is limited. Last but not least, as the data do not document Medicare FFS versus Medicare Advantage plans, we are unable to address the roles of insurance coverage and benefit design, which has serious implications for the future of Medicare telehealth policy.

## Conclusion

Health care providers and policies should aim to reduce barriers to telehealth among older adults, with efforts such as digital literacy support and training. Future research evaluating interventions to improve technology skills among the elderly is warranted.

## Abbreviations Used

NHATS: National Health and Aging Trends Study
SE: standard error

## Notes

^*^
With binary independent variables, marginal effects measure how predicted probabilities change as the binary independent variable changes from 0 to 1. Marginal effects for continuous independent variables measure the amount of change in the dependent variable that will be produced by a 1-U change in the explanatory variable.
